# Mitochondrial Dysfunction in Metabolic Syndrome and Asthma

**DOI:** 10.1155/2013/340476

**Published:** 2013-06-05

**Authors:** Ulaganathan Mabalirajan, Balaram Ghosh

**Affiliations:** Molecular Immunogenetics Laboratory and Centre of Excellence for Translational Research in Asthma & Lung Disease, CSIR-Institute of Genomics and Integrative Biology, Mall Road, Delhi 110007, India

## Abstract

Though severe or refractory asthma merely affects less than 10% of asthma population, it consumes significant health resources and contributes significant morbidity and mortality. Severe asthma does not fell in the routine definition of asthma and requires alternative treatment strategies. It has been observed that asthma severity increases with higher body mass index. The obese-asthmatics, in general, have the features of metabolic syndrome and are progressively causing a significant burden for both developed and developing countries thanks to the westernization of the world. As most of the features of metabolic syndrome seem to be originated from central obesity, the underlying mechanisms for metabolic syndrome could help us to understand the pathobiology of obese-asthma condition. While mitochondrial dysfunction is the common factor for most of the risk factors of metabolic syndrome, such as central obesity, dyslipidemia, hypertension, insulin resistance, and type 2 diabetes, the involvement of mitochondria in obese-asthma pathogenesis seems to be important as mitochondrial dysfunction has recently been shown to be involved in airway epithelial injury and asthma pathogenesis. This review discusses current understanding of the overlapping features between metabolic syndrome and asthma in relation to mitochondrial structural and functional alterations with an aim to uncover mechanisms for obese-asthma.

## 1. Introduction

Mitochondria, dynamic organelles assumed to be originated from *α*-proteobacteria, not only generate energy in the form of ATP but also regulate numerous cellular functions relevant to cell fate, such as apoptosis, generation of oxidative free radicals, and calcium homeostasis [1]. Every mitochondrion has 2 membranous and 2 aqueous compartments: outer membrane, intermembranous space, inner membrane, and matrix [2]. Outer membrane contains numerous porins which form channels through which solutes (≤5000 Daltons) enter freely inside the mitochondria. In contrast, it specifically permits larger mitochondria-targeting signal peptide containing pre-proteins which interact with translocase of outer membrane complex [3]. Mitochondrial intermembranous space, one of the aqueous compartments, contains small molecules which are very similar to cytosol, protein components which vary from cytosol thanks to the restricted entry of larger proteins through outer membrane and protons from oxidative phosphorylation [4]. The inner membrane of mitochondria is folded to form enormous cristae to increase the surface area and to enhance the ATP generating capacity as respiratory chain enzymes that are buried in inner mitochondrial membrane generate ATP by using NADH and FADH2 [5]. Mitochondrial matrix contains the crucial components such as enzymes required for TCA cycle, fatty acid oxidation and pyruvate oxidation, ribosomes, tRNAs, and many copies of mitochondrial genome. Each copy of the mitochondrial genome (16,569 base pairs in human) contains only 37 of its own genes which encode 2 rRNAs, 13 polypeptides, and 22 tRNAs. However, approximately 1500 nuclear encoded proteins are required for proper mitochondrial functions in human [6]. Since various metabolic pathways converge in mitochondrion, it is not surprising to find its involvement in various metabolic diseases including obesity, metabolic syndrome, and hypertension [7]. 

Though the features of metabolic syndrome had been described in 1920s by Kylin, a Swedish physician, only Gerald Reaven had coined the term “Syndrome X” for the collective metabolic abnormalities which were linked with insulin resistance and compensatory hyperinsulinemia [8]. This group of abnormalities is called clinically metabolic syndrome or pathophysiologically insulin resistance syndrome [8]. Later, it was realized that insulin resistance may not be the only dominant metabolic feature as central obesity also leads to most of these metabolic abnormalities independent of insulin resistance [9]. Metabolic syndrome is described as a group of various abnormal metabolic risk factors such as obesity, dyslipidemia, increased blood pressure, increased plasma glucose (prediabetes) levels, prothrombotic condition, and proinflammatory condition [10–12]. These risk factors increase the frequency of cardiovascular diseases, such as heart failure, thrombosis, and cardiac arrhythmias. Most of the patients with metabolic syndrome gradually develop type 2 diabetes and its complications which not only amplify the incidence of cardiovascular diseases but also affect multiple organs causing neuropathies, nephropathies, and so forth [13]. It has been estimated that metabolic syndrome affects 10–30% of the world population [14]. The etiology for metabolic syndrome is complex; genetic, environmental factors and life style are the major assumed etiological components [10–14]. Further, the molecular mechanisms underlying these metabolic risk factors are not clearly known. 

Nevertheless, defective cell metabolism is thought to be one of the main culprits of the syndrome [10]. This defective cell metabolism could be the result of imbalance between nutrient intake and its utilization for energy. Decreased fatty acid oxidation leads to increase in the intracellular accumulation of fatty acyl-CoAs and other fat-derived molecules in various organs such as adipocytes, skeletal muscle, and liver. The accumulated fat molecules inhibit insulin signaling and lead to increase in the levels of insulin in bloodto maintain homeostasis, and this compensatory hyperinsulinemiadamages various organs in metabolic syndrome [10]. Since crucial metabolic pathways ultimately converge in mitochondria, it has been demonstrated that mitochondria become defective in metabolic syndrome. Indeed, it has been referred that metabolic syndrome is a mitochondrial disease [10]. Evidently mitochondrial dysfunction has been demonstrated in various target organs of metabolic syndrome such as adipocyte, skeletal muscle, liver, heart, blood vessels, and pancreatic islet beta cells [15]. However, it is still not clear whether mitochondrial dysfunction is the primary cause or it is the secondary effect of the metabolic syndrome. 

Asthma is generally described by airway hyperresponsiveness, airway inflammation including airway eosinophilia, increased IgE, goblet cell metaplasia, and airway remodeling changes [16]. Though this simple definition is sufficient to explain most of the asthmatic features, it failed to cover various other subtypes of asthma. For example, severe asthma or refractory asthma, which affects 5–10% of all asthmatic patients, is not responsive to currently available medications such as systemic or local corticosteroids [17, 18]. Though severe asthma merely affects less than 10% of asthma population, it consumes significant health resources, contributes significant morbidity and mortality, and dramatically impacts the quality of life [19, 20]. Severe asthma phenotype seems to be beyond the routine definition of asthma, and it requires alternative treatment strategies as well. Various studies have demonstrated that body mass index is positively correlated with asthma severity [21–23]. These indicate that obese-asthma contributes a considerable percentage of severe asthma. In this context, it is imperative to explore the pathogenetic mechanisms of obese-asthma in detail. In this article, we attempted to put together the observations of various studies on the role of mitochondria in the pathogenetic mechanisms for obese-asthma. Though mitochondrial dysfunction is a well-established feature which causes impaired cell metabolism in metabolic syndrome, the role of mitochondria in asthma pathogenesis itself is a relatively new concept [24]. In this context, our lab had shown for the first time the importance of mitochondrial function in asthma pathogenesis [25]. 

In this review article, we discuss the role of mitochondrial dysfunction in causing various risk factors of metabolic syndrome, in airway epithelia of asthmatic airway, and possible role of altered mitochondrial function in lungs of obese-asthma phenotype. Exploring the role of mitochondria in altered lung function is progressively becoming an exciting field of research to understand the pathobiology and treatment of obese-asthma. 

## 2. Mitochondrial Dysfunction in Metabolic Syndrome

### 2.1. Genetic Alterations in Mitochondrial Genome in Patients with Metabolic Syndrome

Metabolic syndrome has not been described as mitochondrial syndrome. However, various studies have reported that genetic alterations in mitochondrial genome are associated with metabolic syndrome. It has been shown that mtDNA/nDNA ratio is drastically reduced in metabolic syndrome [26]. However, this altered ratio has not shown to be associated with any reported genes involved in mitochondrial biogenesis or large mtDNA deletions [26]. Various risk factors of metabolic syndrome have been shown to be associated with T16189C mtDNA variant in both Caucasian and Turkish patients [27, 28]. In a Chinese population, G allele of 10398 A > G mtSNP has been shown to increase the risk of metabolic syndrome [29]. In another study, A3243G mutation in mtDNA has been shown to decrease insulin secretion [30]. Notably, this mutation has also been associated with various mitochondrial diseases, such as myoclonic epilepsy with ragged red fibers, maternally inherited diabetes and deafness [31]. Interestingly, the A3243G mutation which is present in the tRNA-leucine gene causes severe combined respiratory chain assembly defect [32]. Further, thymidine to cytidine mutation present in tRNA-isoleucine has been shown to be associated with hypertension and hypercholesterolemia [33]. In addition, UCP2 promoter polymorphisms are associated with decreased insulin levels and increased prevalence of type 2 diabetes mellitus [34]. 

### 2.2. Mitochondrial Dysfunction Leads to Insulin Resistance (Figure 1)

Even though the mechanism for metabolic syndrome is less clear, it is well established that mitochondrial dysfunction is a critical and common factor associated with almost every feature of metabolic syndrome (see these reviews [7, 13, 15, 33]). It is a surprising paradox that high nutrient intake can lead to decreased oxidative phosphorylation [35]. The imbalance between nutrient intake and its utilization leads to store abnormal lipid in adipocytes and obesity. This accumulated lipid induces various stress pathways and activates various lipid oxidative enzymes. It has been demonstrated that mice which were fed either high fat or western diet had shown the increased expression of 12/15-lipoxygenase (12/15-LOX), a nonheme iron dioxygenase, which catalyzes the hydroperoxidation of polyunsaturated fatty acids, in adipocytes [36]. Further, fat-specific deletion of 12/15-LOX improved glucose metabolism and protected from obesity-mediated complications [37]. 12/15-LOX deficiency not only restored cell metabolism but also decreased the inflammation by reducing the macrophage infiltration in adipose tissue, reducing islet cell inflammation and reducing the circulating proinflammatory cytokines such as IL-6, TNF alpha and increasing anti-inflammatory adiponectin indicating that 12/15-LOX may be essential in causing both local and systemic inflammation in metabolic syndrome and obesity [38]. Increased 12/15-LOX in adipocytes causes ER stress and unfolded protein response which further amplify the stress [39]. Thus, ER stress in adipocytes which is crucial in activating various proinflammatory mediators may be dependent of 12/15-LOX. These evidences indicate that 12/15-LOX could be crucial and essential in the induction of early stages of inflammation in adipose tissue and whole body insulin resistance in high fat fed conditions like metabolic syndrome. The importance of 12/15-LOX in cell fate is evident with its property of direct oxygenation of biomembranes such as mitochondrial membranes even without prior action of phospholipase A2 [40]. Additionally, 12/15-LOX directly depolarizes the mitochondria both *in vitro *and* in vivo* models [41]. In addition, 12/15-LOX may cause mitochondrial dysfunction through its metabolites such as 13-S-hydroxyoctadecadienoic acid (13-S-HODE) and 12-S-hydroxyeicosatetraenoic acid (12-S-HETE) which can cause mitochondrial degradation [42–44]. Though, it has been demonstrated that 12/15-LOX causes insulin resistance in adipocytes [45], the role of mitochondrial dysfunction in that phenomenon is not studied. This could explain the paradoxical reduction in mitochondrial oxidative phosphorylation in adipocytes and other organs with metabolic syndrome. 

**Figure 1:**
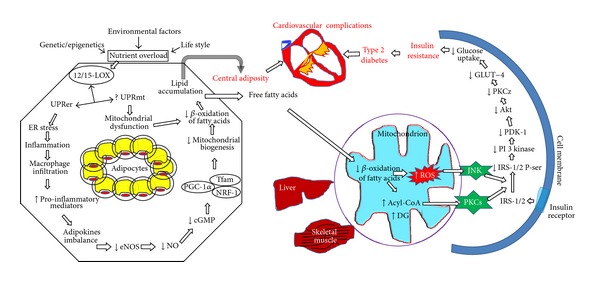
Mitochondrial dysfunction and defective mitochondrial biogenesis lead to insulin resistance and other risk factors of metabolic syndrome. Various etiological factors lead to impair cell metabolism to nutrient overload which increases 12/15-LOX expression in the adipocytes. ER stress and unfolded protein response (UPR) induced by 12/15-LOX increase the adipocyte inflammation and recruits macrophages into the adipocytes. Resultant increase of proinflammatory mediators and imbalance in adipokines reduced eNOS expression. The reduction in eNOS reduces the formation of nitric oxide which impairs mitochondrial biogenesis via cGMP/PGC-1*α* pathway. This decreases beta oxidation of fatty acids and lipid accumulation in adipocytes. The resultant adiposity and release of free fatty acids caused mitochondrial dysfunction and paradoxical reduction in oxidative phosphorylation and increase in the formation of oxidative free radicals. They further activate JNK and PKCs which cause serine phosphorylation of IRS-1/2 leading to insulin resistance by decreasing PI3-K/PDK-1/Akt signaling. This causes the development of type 2 diabetes which along with central adiposity amplifies the risk of cardiovascular diseases in metabolic syndrome. 12/15-LOX: 12/15-lipoxygenase; UPRer: unfolded protein response in endoplasmic reticulum (ER); UPRmt: unfolded protein response in mitochondria; eNOS: endothelial nitric oxide synthase; cGMP: cyclic guanosine monophosphate; PGC-1*α*: peroxisome proliferator-activated receptor gamma coactivator 1 alpha; NRF-1: nuclear respiratory factor-1; Tfam: mitochondrial transcription factor; DG: diacylglycerols; ROS: reactive oxygen species; PKC: protein kinase C; JNK: c-Jun NH(2)-terminal kinase; IRS, insulin receptor substrates; PI3 kinase: phosphoinositide 3-kinase; PDK-1: phosphoinositide-dependent kinase-1.

Increased ER stress and its consequent unfolded protein response (UPR), possible mitochondrial dysfunction, and resultant oxidative stress along with increased accumulation of macrophages lead to the release of proinflammatory mediators such as TNF-*α* and IL-1β from adipocytes ([13, Figure 1]). These mediators reduce eNOS expression in adipocytes. The reduction in eNOS expression is followed by decreased NO production and reduced mitochondrial biogenesis by inhibiting cGMP and PGC-1*α* (peroxisome proliferator-activated receptor coactivator 1 alpha) which is the master regulator in mitochondrial biogenesis. The eNOS deficient mice showed a group of metabolic abnormalities found in metabolic syndrome patients such as dyslipidemia, hypertension, and insulin resistance [46]. Further, the reduction in mitochondrial biogenesis jeopardizes the beta oxidation of fatty acid and thus leads to accumulation of lipids in adipocytes to cause lipid overload in adipocytes. This increased ER stress in adipocytes leading to release of various proinflammatory mediators which further reduce eNOS expression to complete the vicious cycle (Figure 1). This leads to disabling the mitochondriogenesis and amplifying the features of visceral obesity which is an initiating factor for the development of most of the risk factors of metabolic syndrome. These evidences indicate the crucial role of eNOS in metabolic syndrome in the aspect of mitochondrial biogenesis [47, 48]. 

The resultant central obesity leads to release of free fatty acids (FFA) in plasma and these (Figure 1) FFAs damage various organs such as liver, skeletal muscle, pancreatic beta islet cell, and blood vessels [49–51]. This leads to the reduction in the metabolic function of mitochondria and glucose uptake with increase in glucose synthesis and insulin secretion to cause a reduction in insulin responsiveness [49–52]. These indicate that mitochondrial dysfunction is crucial not only in causing insulin resistance but also in developing other risk factors of metabolic syndrome such as hypertension and type 2 diabetes mellitus. 

### 2.3. Defective Mitochondrial Biogenesis in Metabolic Syndrome

It has been hypothesized that mitochondrial dysfunction and mitochondrial biogenesis are interrelated though molecular components for these processes are somewhat different. In many diseases, it has been demonstrated that mitochondrial biogenesis compensates the mitochondrial dysfunction [53, 54]. In contrast, mitochondrial dysfunction observed in various organs of metabolic syndrome is associated with a reduction of factors that are necessary for mitochondrial biogenesis. This could be due to two possible reasons: (a) the possibility of increased severity of mitochondrial dysfunction and (b) defective mitochondrial biogenesis [13]. The reduction of mitochondrial biogenesis that occurs in adipocytes is one of the crucial events in initiating metabolic syndrome as shown in Figure 1. However, the reduction in mitochondrial biogenesis is not only restricted to adipocytes but occurs in various other organs, like cardiomyocytes, liver, skeletal muscle, and so forth [55, 56]. 

### 2.4. Role of Mitochondrial Sirtuins in Metabolic Syndrome

The sirtuins, nicotinamide adenine dinucleotide-dependent protein deacetylases, regulate various processes of cellular metabolism. There are 7 sirtuins (silent mating type information regulation 2 homolog) characterized so far in human (SIRT1-SIRT7) [57]. Amongst all, SIRT3-5 are localized in mitochondria and deacylate various crucial enzymes to regulate mitochondrial function [58]. SIRT3 deacetylates various key enzymes such as long-chain acyl-CoA dehydrogenase. SIRT3-mediated deacetylation leads to increase in mitochondrial fatty acid oxidation in liver whereas its deficiency leads to metabolic syndrome like features by mitochondrial protein hyperacetylation [59]. High-fat diet increases the mitochondrial protein hyperacetylation in liver along with the reduction in SIRT3 [60]. While PGC-1*α* is known to be downregulated in metabolic syndrome, its activation leads to mitochondrial biogenesis. On the other hand, PGC-1*α* regulates SIRT3 gene expression. This indicates that fasting or calorie restriction could reverse the features of metabolic syndrome by activating both SIRT3 and PGC-1*α* which increase fatty acid oxidation and mitochondrial biogenesis, respectively. Interestingly, SIRT1 positively regulates SIRT3 through activating PGC-1*α*. Importantly, transgenic Sirt1 mice had shown resistance in developing metabolic syndrome [61]. 

## 3. Mitochondrial Dysfunction in Asthma

### 3.1. Genetic Alterations in Mitochondrial Genome of Asthmatic Patients

Asthma is not considered as a mitochondrial syndrome as there is no consistent report to demonstrate the mitochondrial DNA mutations in asthmatic patients. However, there is a considerable overlap between asthma pathophysiology and mitochondrial biology in the aspects of oxidative stress, apoptosis, and calcium ion homeostasis. These overlaps are mostly related to mitochondrial functions which indicate that the observations of mitochondrial dysfunction in asthma are most likely due to secondary effects rather than its causal effects. However, maternal inheritance is considered as one of the strongest risk factors of asthma and other atopic diseases [62–65]. Hence, mitochondria are suggested to be involved in the vertical transmission of asthma. Also, mitochondrial haplogroups have been shown to be associated with increased serum IgE levels in European population [65, 66]. Further, various mutations in mitochondrial genes encoding mitochondrial tRNAs have been reported to be associated with asthma [66]. In addition, ATP synthase mitochondrial F1 complex assembly factor 1 gene is found to be associated with asthma in Caucasian European children [67]. Very interestingly, A3243G tRNALeu (UUR) functional mutation was found to be present in some rare forms of asthma which is also associated with hypertension, ischemic heart disease, and age-related maculopathy [68]. These evidences of genetic association indicate a possible causal effect of mitochondria in asthma rather than a secondary effect. 

### 3.2. Mitochondrial Dysfunction in Asthmatic Airway Epithelia

Earlier, it was believed that most of the asthma features are mediated by Th2 cytokines [69]. However, the exact role of airway epithelia in asthma pathogenesis was not studied and indeed it was neglected assuming that the airway epithelium is a target cell type for infiltrating immune cells. However, recent genetic and functional evidences indicated the central role of airway epithelia in lung homeostasis [70, 71]. The airway epithelia maintain airway homeostasis by secreting various anti-inflammatory mediators and bronchodilators in normal airway. This homeostasis is disturbed in case of epithelial injury. For example, stressed epithelia secrete critical cytokines such as IL-33, IL-25, and thymic stromal lymphopoietin which lead to Th2 polarization [72–74]. Thus, it seems that airway epithelia act as a governing factor to decide the status of inflammation in the airway, thus maintaining airway epithelial homeostasis which is essential for proper lung function. 

Hence, the mechanisms for epithelial injury were not studied in detail due to the belief that epithelial injury could be due to inflammation. However, there were scattered evidences to indicate the possible role of mitochondrial dysfunction in airway epithelial injury in asthmatic conditions. The ultrastructural observations of human asthmatic bronchial epithelium showed swollen mitochondria in 1985, and similar observations were made in mouse model of asthma later [75, 76]. However, a detailed structural and functional study on mitochondrial abnormality was first reported by our group in asthmatic mice [25]. We demonstrated that asthmatic mice lungs had a reduction in the expressions of cytochrome c oxidase (third subunit) and complex I (17 kDa subunit) in bronchial epithelium, loss of cristae with mitochondrial swelling in bronchial epithelium, decreased cytochrome c oxidase activity in lung mitochondria, increased cytochrome c in lung cytosol, and a reduction in lung ATP levels [25]. Further, we have demonstrated that various pharmacological compounds such as baicalein, esculetin, vitamin E, resveratrol, simvastatin and thionocinnamates restore mitochondrial dysfunction, and thus attenuate asthma features [77–82]. We further demonstrated that linoleic acid metabolite, 13-S-HODE, can cause mitochondrial dysfunction in airway epithelia to drive severe asthma by activating transient receptor potential vanilloid type 1 (TRPV1) [42]. Importantly, 13-S-HODE administration to naïve mice leads to significant neutrophilia, difficulty in breathing, and airway injury. As 13-S-HODE is found to be increased in asthmatic airways these findings have clinical importance [42]. In another study, mice that had the deficiency of mitochondrial ubiquinol-cytochrome c reductase core II protein in airway epithelium aggravated asthma features [83]. In addition to these mouse studies, mitochondrial dysfunction had been observed in human asthmatic bronchial epithelia [84]. These evidences indicate that adequate function of mitochondria is essential to maintain the epithelial health, and studies focusing on epithelial biology could be therapeutically beneficial. Further, detailed studies are required to understand the molecular mechanisms for the observed mitochondrial dysfunction in asthmatic airway epithelia. The imbalance between oxidative stress and antioxidants in inflammation-mediated oxidative microenvironment may lead to these changes. Evidently, superoxide dismutase has been shown to be reduced in asthmatic airway [85, 86]. In contrast to this view, few studies have demonstrated the possible causative role of mitochondrial dysfunction in asthma pathogenesis. For example, increased mitochondrial respiratory complex III mediated ROS production amplified Th2 responses [87]. In addition, preexisting mitochondrial dysfunction in airway epithelia worsened asthma features [83]. These evidences along with the association of mitochondrial haplogroups in asthma also suggested the possible causal role of mitochondrial dysfunction in asthma pathogenesis. Thus, more detailed studies are required to understand the causal role of mitochondrial dysfunction in airway epithelial injury of asthmatics. 

### 3.3. Mitochondrial Biogenesis in Asthmatic Airway Smooth Muscle

The bronchial epithelial injury in asthmatic airway activates epithelial mesenchymal trophic unit that increases various growth factors such as TGF-beta, FGF, and VEGF leading to airway remodeling which consists of epithelial and hyperplasia and hypertrophy, goblet cell metaplasia, increased airway collagen deposition, hypertrophy and hyperplasia of airway smooth muscle [88, 89]. Among these features, hyperplasia and hypertrophy of airway smooth muscle and subepithelial fibrosis are crucial components. Though asthmatic airway epithelia have the dysfunctional mitochondria, increased number of mitochondria was observed in asthmatic bronchial smooth muscle due to increase in the expression of key proteins involved in mitochondrial biogenesis [90]. Further, it has been shown that mitochondrial biogenesis may be essential to cause smooth muscle hypertrophy involved in airway remodeling. So it appears that status of mitochondria may be different in different cell types of asthmatic lungs. 

## 4. Overlapping Mitochondrial Features between Metabolic Syndrome and Asthma: A Possible Role of Mitochondria in the Pathogenesis and Therapeutics of Obese-Asthma (Figure 2)

### 4.1. Obese-Asthma: A Distinct Clinical Asthma Phenotype

Mitochondrial dysfunction and defective mitochondrial biogenesis in various organs such as adipose tissue, muscle, liver, and pancreatic beta islet cell and vessel are known in metabolic syndrome [7, 13, 15, 33]. Also, the involvement of mitochondria in metabolic syndrome is theoretically obvious as mitochondrion is a converging point for various cellular metabolic pathways. However, the causal role of mitochondria in the risk factors associated with the features of metabolic syndrome remains to be explored. On the other hand, the involvement of mitochondria in asthma pathogenesis is relatively new and has not been explored in details [25, 42, 77–84]. Obese-asthma, a distinct clinical phenotype of asthma, has been characterized with the presence of neutrophilic airway inflammation, nonatopic nature, low-grade systemic inflammation, increased morbidity, and being resistant to corticosteroids [91–99]. Due to these differences, pathophysiology of obese-asthma may not be similar to prototype asthma and thus needs to be explored for developing effective alternative treatments. Towards this effort, it would be useful to understand the possible overlapping mechanisms between metabolic syndrome and asthma. Earlier, it was believed that these two diseases are just coincidental and might share few comorbid features. However, innate airway hyperresponsiveness in obese mice even without allergen immunization, development of severe asthma to common asthma predisposing factors, and improvement of asthma in patients who lose weight suggest that there could be a causal relationship between obesity and asthma rather than simple coincidence [100, 101]. Thus, exploring the overlapping mechanisms between obesity and asthma could open new therapeutic avenues for obese-asthma, a severe and steroid resistant form of asthma.

 Recently, increased expression of certain proinflammatory mediators such as leptin, IL-6, TNF-*α*, and C-reactive protein and decreased expression of adiponectin have been demonstrated in obese-asthmatics [102]. This alteration of cytokines and adipokines may play an important role in pathogenesis of obese-asthma. However, the interplay between these two diseases may not be a simple coincidence but there could be due to bidirectional and complex molecular interactions. For example, obese-asthmatics are more prone to develop certain riskfactors of metabolic syndrome compared to obese-nonasthmatics which indicates that asthma could potentiate the risk of developing metabolic syndrome [103]. Similarly, metabolic syndrome could potentiate the asthma severity [96, 98]. However, due to the lack of available literature in this area, it is premature to speculate further and we need to collect enough data related to experimental and epidemiological studies. 

### 4.2. 12/15-LOX, eNOS, and ADMA: Overlapping Mitochondrial Features between Metabolic Syndrome and Asthma (Figure 2)

As described earlier, 12/15-LOX seems to be the crucial proinflammatory lipid peroxidative enzyme in initiating metabolic syndrome, and importantly 12/15-LOX deletion had shown to be beneficial in the reduction of the features of metabolic syndrome by reducing ER stress in adipocytes [36–39, 45]. 12/15-LOX degenerates mitochondria present in the reticulocyte in the process of RBC maturation as it acts directly on phospholipid esters present in mitochondrial membrane [104]. Further, 12/15-LOX metabolites such as 13-S-HODE cause mitochondrial dysfunction in airway epithelia in asthma pathogenesis by activating TRPV1 [42]. On the other hand, the importance of 12/15-LOX is well known in asthma pathogenesis as its genetic deletion alleviates asthma features [105, 106]. These indicate that 12/15-LOX seems to be an attractive target in obese-asthmatics. 

**Figure 2:**
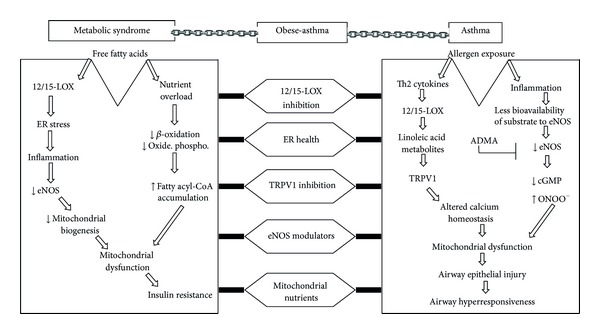
Overlapping mitochondrial features between metabolic syndrome and asthma. A possible role of mitochondria in the pathogenesis and therapeutics of obese-asthma. Common factors between metabolic syndrome and asthma in the aspect of mitochondrial dysfunction may be used as therapeutic targets in obese-asthma. In asthma, the infiltrated inflammatory cells increase 12/15-LOX which secretes linoleic acid metabolite (13-S-HODE) which causes mitochondrial dysfunction by activating TRPV1 that disturbs calcium homeostasis and increases mitochondrial calcium overload to cause mitochondrial dysfunction. On the other hand, inflammation leads to increase in the expressions of arginase and iNOS which consume L-arginine to cause less bioavailability of L-arginine to eNOS. Further, increased ADMA uncouples eNOS to generate more ROS and peroxynitrite which cause mitochondrial dysfunction. The resultant mitochondrial dysfunction in airway epithelia leads to injure airway epithelia and causes airway hyperresponsiveness. Most of the sequences of increased free fatty acid in metabolic syndrome have been explained in Figure 1. Thus, 12/15-LOX inhibition, improving ER health, TRPV1 inhibition, increased eNOS, and mitochondrial nutrients could be possible therapeutic targets in obese-asthmatic condition. TRPV1: transient receptor potential cation channel, subfamily V, member 1; ONOO^−^: peroxynitrite.

Reduced bioavailability of endogenous L-arginine seems to the common pathophysiological feature in both asthma pathogenesis and metabolic syndrome [107–109]. eNOS has a protective role in both diseases [110–112] whereas ADMA, an endogenous inhibitor of NO that uncouples eNOS to generate more oxygen-free radicals. ADMA is found to produce harmful effects on obesity, metabolic syndrome, and asthma as increased ADMA reduces arginine bioavailability to eNOS [107, 108, 113, 114]. This imbalance between ADMA/L-arginine could lead to endothelial dysfunction which is one of the common denominator for various cardiovascular diseases and insulin resistance [113]. It is also to be noted that reduction in the endogenous bioavailability of nitric oxide also reduces mitochondrial biogenesis which could lead to reduced fatty acid oxidation. Thus, improving the bioavailability of NO by ADMA inhibition could lead to improve both endothelial dysfunction and insulin resistance. Similar metabolic alteration in NO related to mitochondrial dysfunction has been demonstrated in asthmatic lungs [108, 114, 115]. In normal airway, eNOS present in the healthy epithelia generates low levels of NO to maintain airway tone by activating soluble guanyl cyclase and cGMP production. This homeostatic process in the airway epithelia is jeopardized in asthmatic airway as reduced bioavailability of L-arginine to eNOS due to increase in the levels of competitors such as arginase, inducible NOS which also consumes L-arginine. Also, asthmatic airway was found to have increased ADMA which not only decreases the bioavailability of L-arginine to eNOS but also uncouples eNOS to generate reactive oxygen-free radicals to generate more peroxynitrite which is a powerful bronchoconstrictor. Interestingly, eNOS-deficient mice had shown the features of metabolic syndrome, and on the other hand eNOS overexpression in bronchial epithelial alleviated asthma features [116]. In addition, high dose of L-arginine supplementation had shown to alleviate asthma features and metabolic syndrome [108, 109] in independent studies. Thus, enhancing bioavailability of endogenous L-arginine by various approaches such as exogenous L-arginine supplementation, ADMA inhibition, and overexpression of eNOS could improve mitochondrial dysfunction by increasing mitochondrial biogenesis and reducing the generation of peroxynitrite (Figure 2). Hence, strategies can be formulated to modify NO pathway to improve the fatty acid oxidation and to increase the bronchorelaxation for obese-asthma phenotypes by targeting eNOS and ADMA. Importantly, all of these approaches are independent of Th1/Th2 paradigm as obese-asthma, and other forms of severe asthma do not follow Th1/Th2 paradigm, and indeed increased IFN-*γ* has been shown to be essential in development of severe asthma [117]. 

### 4.3. Missing Mitochondrial Links in Obese-Asthma

Since mitochondrial dysfunction seems to be the common denominator between metabolic syndrome and asthma, more studies can focus on exploring the exact role of mitochondria in obese-asthmatics. While it is known that various organs affected with metabolic syndrome are actively involved in glucose metabolism, the glucose metabolism in the lungs was not studied in detail and the diseases which affect predominantly lungs due to metabolic diseases are also not known. It seems that bronchial epithelium is an active cell type which maintains airway homeostasis [70, 71]. As highly energy-dependent cells express more of 17 kDa subunit of complex I, its predominant and exclusive expression on bronchial epithelia indicates its active role in metabolic pathways [25, 118]. Thus, it would be interesting to know whether mitochondrial dysfunction in airway epithelia could lead to insulin resistance and affects-lung homeostasis. Similarly, effects of hyperinsulinemia on lung homeostasis and airway epithelial injury need to be explored in obese-asthmatics. Since mitochondrial dysfunction has been reported in skeletal muscle of metabolic syndrome, it would be interesting to explore the status of mitochondria in airway smooth muscle of obese-asthmatics. In this context, mitochondrial biogenesis has been reported in airway smooth muscles of asthmatics and suggested its role in causing airway remodeling. 

### 4.4. Mitochondria-Targeted Therapeutics for Obese-Asthma (Figure 2)

As various reports have explored the beneficial role of mitochondrial targeted molecules independently both in metabolic syndrome and asthma, mitochondria-targeted pharmaceutical targets would be attractive targets in obese-asthmatics. Various natural compounds that potentiate mitochondrial biogenesis protect mitochondria from oxidative damage termed as mitochondrial nutrients [119]. They are coenzyme Q, *α*-lipoic acid, acetyl L-carnitine, *α*-tocopherol, glutathione, creatine, pyruvate, and choline [119]. Among these mitochondrial micronutrients, many of them have shown the beneficial effects both in metabolic syndrome and asthma in various independent studies [120–123]. For example, Coenzyme Q10, mitochondria-targeted antioxidant, has shown the beneficial effect in reducing corticosteroid dosage in asthmatics [120]. Similarly, Coenzyme Q10 administration prevented hyperinsulinemia, improved the endothelial dysfunction, and reduced hypertension along with the reduction in the increase of oxidative and nitrative inflammatory markers in rat model of metabolic syndrome [121]. Further, Coenzyme Q10 recouples eNOS to reduce the formation of oxidative free radicals and improve endothelial dysfunction and mitochondrial oxidative phosphorylation to attenuate the features of diabetic endotheliopathy [121]. *α*-tocopherol has been shown to reduce the mitochondrial dysfunction in asthma pathogenesis [79]. Similar beneficial effects of *α*-tocopherol have been reported in metabolic syndrome [123]. Mitochondria-targeted antioxidant, MitoQ, prevented adiposity, hyperglycemia, hypercholesterolemia, and hypertriglyceridemia and hepatic steatosis in fat fed ApoE-deficient fat fed model of metabolic syndrome [124]. While it is known that *α*-lipoic acid increased the expression of PPAR-*γ* in cardiomyocytes and beneficial in metabolic syndrome, *α*-lipoic acid also has antiasthma property [125, 126]. It is to be noted that PPAR-*γ*/PGC-1*α* pathway improves mitochondrial bioenergetics [127]. Resveratrol has been shown to improve mitochondrial function both in asthmatic models and metabolic syndrome models [80, 128, 129] whereas resveratrol activates SIRT-1 [130]. While SIRT-1 is the pharmacological target in metabolic syndrome as it induces mitochondrial biogenesis by activating PGC-1*α*, SIRT-1 activator (SRT1720) reduced the features of allergen induced airway inflammation [131]. Resveratrol is also known to increase the expression of inositol polyphosphate-4-phosphatase (INPP4A) [80]. Moreover, INPP4A gene variants were found to be associated with metabolic syndrome and asthma, and very interestingly INPP4A is also one of the insulin-signaling molecules [132–134]. Though various antioxidants have shown the beneficial effects in preclinical studies, most of the antioxidants have poor therapeutic success in various clinical trials. This could be due to the poor distribution of various available antioxidants to different organs and different cellular compartments such as mitochondria which are the major sources of reactive free radicals [135–137]. Thus, direct targeting of antioxidants to mitochondria could prove to be beneficial in future clinical trials. 

## 5. Conclusions and Future Perspective 

Mitochondrial dysfunction and defective mitochondrial biogenesis in various organs such as adipose tissue, muscle, liver, and pancreatic beta islet cell and vessel are known in metabolic syndrome. On the other hand, the involvement of mitochondria in airway epithelial injury and asthma pathogenesis has been recently demonstrated. Mitochondrial dysfunction seems to be common denominator for the risk factors of metabolic syndrome and airway epithelial injury in asthma. Thus, exploring the overlapping mechanisms between obesity and asthma could open new therapeutic avenues for obese-asthma, a severe and steroid resistant form of asthma. 12/15-LOX, eNOS, and ADMA are few overlapping features between metabolic syndrome, and asthma (Figure 2) in the aspect of mitochondrial dysfunction and thus more studies can be initiated to explore this further. In conclusion, protecting mitochondria seems to be an attractive therapeutic strategy in obese-asthmatic condition.
